# Ten years of Pan-InfORM: modelling research for public health in Canada

**DOI:** 10.3934/publichealth.2021020

**Published:** 2021-03-15

**Authors:** Mehreen Tariq, Margaret Haworth-Brockman, Seyed M Moghadas

**Affiliations:** 1 Agent-Based Modelling Laboratory, York University, Toronto, ON, Canada; 2 National Collaborating Centre for Infectious Diseases, University of Manitoba, Winnipeg, MB, Canada; 3 Department of Community Health Sciences, University of Manitoba, Winnipeg, MB, Canada

**Keywords:** disease modelling, public health, knowledge translation, community of practice, model-based policy, emerging infectious diseases

## Abstract

Modelling and simulation methods can play an important role in guiding public health responses to infectious diseases and emerging health threats by projecting the plausible outcomes of decisions and interventions. The 2003 SARS epidemic marked a new chapter in disease modelling in Canada as it triggered a national discussion on the utility and uptake of modelling research in local and pandemic outbreaks. However, integration and application of model-based outcomes in public health requires knowledge translation and contextualization. We reviewed the history and performance of Pan-InfORM (Pandemic Influenza Outbreak Research Modelling), which created a national infrastructure in Canada with a mandate to develop innovative knowledge translation methodologies to inform policy makers through modelling frameworks that bridge the gaps between theory, policy, and practice. This review demonstrates the importance of a collaborative infrastructure as a “Community of Practice” to guide public health responses, especially in the context of emerging diseases with substantial uncertainty, such as the COVID-19 pandemic. Dedicated resources to modelling and knowledge translation activities can help create synergistic strategies at the global scale and optimize public health responses to protect at-risk populations and quell socioeconomic and health burden.

## 1. Introduction

Public health crises, particularly outbreaks of emerging infectious diseases, are inevitable and often demand a population-wide effort for containment. Without timely implementation of adequate control measures, disease outbreaks can lead to tremendous social and economic burden, as we see with COVID-19. However, rapid decision-making to curtail disease spread and protect populations is challenging, especially when there is limited knowledge and substantial uncertainty about a new disease and the potential outcomes of public health decisions. Public health professionals require evidence and data to determine efficient, effective and economically feasible interventions. Foundational to this requirement is successful collaboration between knowledge generators (e.g., researchers, health professionals) and knowledge users (e.g., planners, providers, decision-makers). Knowledge generation requires appropriate methods, resources and expertise to obtain valid and reliable results that can be contextualized. Concomitantly, knowledge users require clarity about assumptions, the research methods used, how to interpret results, and any limitations on how to apply conclusions in real-world situations.

Mathematical and simulation models provide a complementary approach for knowledge generation, especially for informing public policy. Models provide a means to analyze and validate different hypotheses about multifaceted disease and population systems and to understand underlying interdependencies. Models can also be used to compare different interventions and to project possible outcomes, including best- and worst-case scenarios. Policy makers can use model projections to reduce uncertainty about the potential effects of their decisions. In this context, effective collaboration between research modellers and policy makers is fundamental for designing, implementing and optimizing control policies.

The Pandemic Influenza Outbreak Research Modelling (Pan-InfORM) team of modellers and public health practitioners was established in Canada with a mandate to “develop innovative knowledge translation methodologies and inform policy makers through modelling frameworks that forge strong links between theory, policy, and practice” [1]. While its networking activities began in the fall of 2008, Pan-InfORM was formally established during the early stages of the 2009 H1N1 influenza pandemic, assessing mitigation strategies and providing guidance to public health planners and decision makers to optimize health responses [1]. In this ten-year review, we report on the research activities of Pan-InfORM, its key achievements, and its contributions to public health in Canada and around internationally.

The underlying principles used in the establishment of this national network are derived from the “Communities of Practice” (CoP) model [2],[3]. Since the earliest human history, the communities of practice model would simply be “knowledge-based social structures” where individuals with a common goal would interact and offer their expertise [3]. This simple CoP model can be used to connect experts globally to exchange knowledge and experience, and identify strategies to overcome disciplinary and multi-disciplinary challenges [3]. Due to the dynamic nature of knowledge generation, a CoP would promote the standardization of terms underpinning well-established concepts and allow members to direct their efforts to areas with a greater degree of uncertainty [1],[3].

## 2. The genesis of Pan-InfORM

Prior to the 2003 SARS epidemic, modelling studies in Canada were typically focused on epidemic theory and disease dynamics, perhaps due to limited communication and collaboration between disease modellers and policymakers. During the SARS epidemic, modelling and simulation tools were used to understand and estimate important model factors, such as the reproduction number [4]. Model estimates helped predict the epidemic trajectory and identify the most effective interventions. The results also highlighted the potential for modelling studies to inform public health policy, sparking interest in both modellers and public health professionals who recognized the significance of modelling in real-world applications.

Following the SARS epidemic, several events were organized to establish communication between modellers and policy makers. Two workshops held by Public Health Agency of Canada in 2006 and 2007 focused on understanding the optimal use of antiviral drugs and vaccines. These early networking sessions highlighted the abundance of skills and expertise available in Canada; however, they also shed light on significant barriers that hindered uptake of modelling results by policy makers. Effective translation of modelling studies to support decision-making and public health actions required some new infrastructure. Pan-InfORM's national network of modelling and public health leaders was established to help bridge knowledge translation gaps while conducting research to guide important public health decisions.

**Figure 1.**
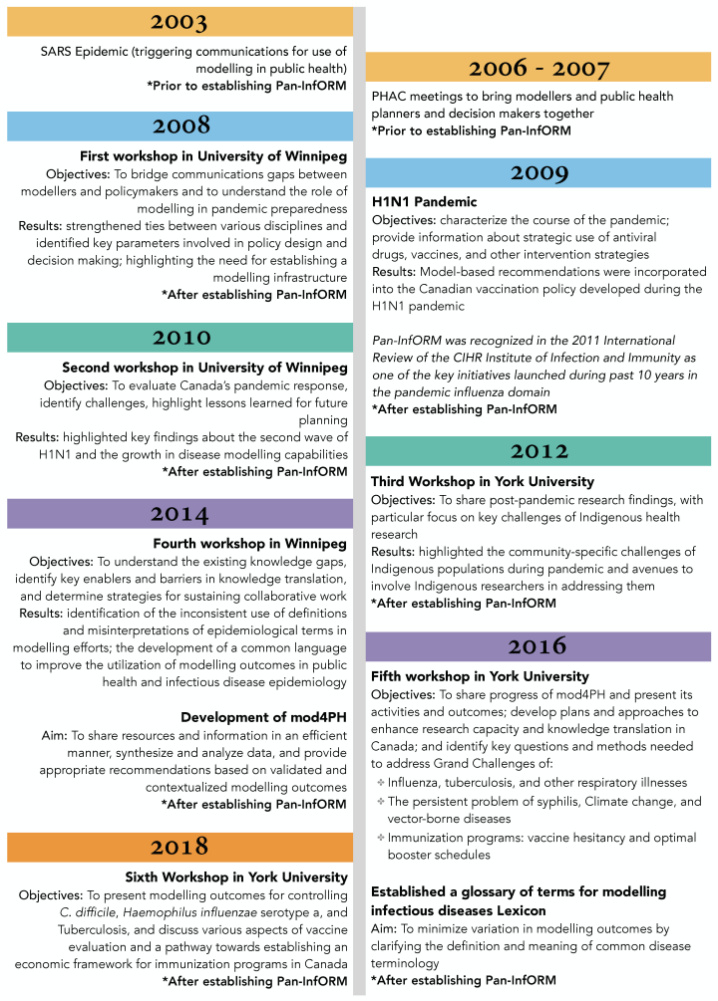
Timelines of Pan-InfORM activities and their outcomes.

Figure 1. illustrates the progression of Pan-InfORM's networking workshops and modelling research over 10 years. For the most part, workshops were held every second year, giving time for modellers and public health practitioners to undertake new projects between meetings. The workshops were opportunities to reconvene, bring in new stakeholders and discuss progress as well as new knowledge gaps and opportunities.

## 3. The role of modelling in pandemic responses

Pan-InfORM held its first international workshop in 2008 at the University of Winnipeg, Manitoba. The primary objectives were to bridge communication gaps between modellers and policymakers and to understand the role of modelling in pandemic preparedness. Modellers presented their evaluations of preventative, therapeutic, and social distancing measures [5]. In the ensuing discussions four themes emerged, concerning research efforts to: i) minimize serious illness, deaths, and societal disruption during a pandemic; ii) increase pandemic vaccine lead time using strong surveillance systems; iii) increase immunization rates among children as significant transmitters of disease; and iv) identify strategic use of antiviral therapy prior to pandemic vaccine availability [5]. During the workshop, it became evident that while modelling research in Canada was at the forefront of pandemic preparedness, effective disease management requires integrating modelling research and outcomes in public health actions. Participants noted that models should be used to help design and select appropriate interventions while considering important health, economic (i.e., cost-effectiveness) and ethical factors. An essential component of the modelling framework was the support from healthcare systems (e.g., surveillance and data) and government organizations (e.g., funding) [5].This support should also provide the necessary resources and information (such as access to data, hosting information sessions, developing channels for direct communications between researchers, planners and policymakers, and identifying key questions, assumptions and priority areas for research) to develop data-driven models and generate evidence-based outcomes for rapid responses in times of crises [5].

The first influenza pandemic of the 21st century, H1N1, emerged in 2009. Canadian modellers, including the Pan-InfORM team, directed research efforts to investigate the characteristics and dynamics of the disease [1],[6],[7]. The team's efforts provided health practitioners with information about risk of hospital admission and case-fatality rates for different age groups [7]. Support from the Canadian Institutes of Health Research (CIHR) allowed Pan-InfORM to also provide valuable insight on the strategic use of antiviral drugs, vaccines, and other intervention measures [7]. Pan-InfORM members' diverse expertise, strong partnerships and effective knowledge translation methods helped to optimize public health responses. These achievements were highlighted in an international review by the CIHR on pandemic research in 2011 [8].

After the H1N1 pandemic, Pan-InfORM held its second public health workshop in 2010, to evaluate the role of modelling studies in Canada's pandemic response and to share research progress and challenges. Participants presented studies on intervention strategies and disease epidemiology, especially in Indigenous communities. Presenters reported examples where modelling research was used to guide pandemic response, such as the development of vaccine strategies to prevent severe outcomes and vaccine cost-effectiveness analysis [9]. As new information emerged during the initial stages of the 2009 pandemic, the Public Health Agency of Canada made a number of amendments to antiviral drug recommendations on the basis of modelling and cost-effectiveness studies. Follow-up discussions at the 2010 workshop suggested that models should be combined with operations research to further optimize resource allocation and identify frameworks to set priorities for vaccine delivery in different provinces [9]. Discussions on social distancing measures, such as school closures, highlighted the need to integrate economic factors (e.g. costs incurred to families associated with closing schools) in models for an in-depth analyses of these strategies and their effects on the community [9].

Over the course of the workshop, the organization of disease surveillance in Canada was identified as an area for improvement and coherence. Important information required for appropriate responses was not readily available or there were discrepancies and deficiencies in data collection. These inconsistencies affect estimates of disease characteristics, model parameters, and public health decisions. Workshop participants recommended that standardized surveillance and linkable databases across the country would improve capacity to collect and analyze large data sets during an evolving epidemic. Although there had been tremendous growth in pandemic modelling since the 2008 workshop, participants noted there were still some uncertainties and gaps in knowledge which called for further investigation [9].

## 4. Initiatives to protect vulnerable populations

Following the 2010 workshop, Pan-InfORM team members conducted further research on the characteristics of the H1N1 pandemic and the impact on Canadian communities, including on transmissibility of the disease [10],[11] evaluation of intervention strategies [12], and disease spread in remote and isolated communities [13]. Many communities, in northern Manitoba for example, were disproportionately affected by H1N1 pandemic. The risk of hospitalization and intensive care unit admission among some First Nations communities was three times higher than for non-First Nations populations [14]. Some members of Pan-InfORM developed an agent-based model of a small Indigenous community to understand the dynamics of influenza transmission and the impact of age, household size, and pre-existing immunity on disease burden in isolated communities [13].

Pan-InfORM held its third workshop in 2012 to share post-pandemic research findings, and to focus on critical aspects of research with Indigenous communities. Workshop presentations and discussions highlighted key post-pandemic research findings: (i) the disproportionate effects seen in Indigenous communities of Manitoba and Nunavut; (ii) efforts made to improve communication between First Nations and Métis self-governments and communities with federal and provincial governments to evaluate and assess their access to vaccines; (iii) assessment of strategies to distribute flu-kits and station health care teams in isolated areas; (iv) the significance of geography as a determinant of disease severity as many underserved communities were remote and isolated, and; (v) an observed lack of consistency because organizations and communities developed their own pandemic response plans [14]. For instance, the workshop summary mentioned that some communities in Nunavut experienced cultural barriers, such as residents experiencing hesitancy with healthcare practitioners, considering them as “outsiders” [14]. Furthermore, one study presented during the workshop found that although residents of Montreal and First Nations reserve Kahnawà:ke had close access to healthcare services, and the residents experienced 33% higher outpatient and emergency room visits, highlighting their stark vulnerability to emerging diseases [14]. In general, better knowledge and understanding of community-specific challenges, including cross-cultural and logistical barriers, were recognized as key when analyzing research findings. The idea of a CoP to increase collaboration between researchers and Indigenous stakeholders and to inform modelling research on population-specific disease parameters was highlighted [14].

Following this workshop, some members of the Pan-InfORM network conducted critical research on the “Optimal Treatment Strategies for Remote and Isolated Communities”, supported by the Public Health Agency of Canada [15]. Specifically, the team developed an age-specific mathematical model to evaluate attack rates of potential influenza pandemic strains and understand the effects of different post-exposure prophylaxis treatment levels on influenza dynamics in isolated communities [15].

## 5. Modelling for public health: Mod4PH

A 2014 study by Pan-InfORM members documented the reoccurring factors needed for model-based policies: (i) considering public health priorities, (ii) communicating the model's assumptions and limitations, and (iii) involving decision-makers in the model construction process [16]. Based on these findings, Pan-InfORM recognized that the formation of a successful CoP would require effective collaboration amongst members with diverse expertise, “jargon-free” terminology and bi-directional communication to inform subsequent research activities [2],[16].

At the fourth Pan-InfORM workshop in 2014, held in partnership with the National Collaborating Centre for Infectious Diseases (NCCID) and the International Centre for Infectious Diseases (ICID), a primary objective was to determine strategies for sustained collaborative work in modelling for public health [17]. Public health members pointed out decision-making is multi-faceted, often involving input from different levels of governments. The involvement of policymakers and end-users in the model development process allows them to gain an explicit understanding of the capability and limitation of models as well as the interpretation of the results.

Discussions also centred on the inconsistent use of definitions and misinterpretations of epidemiological terms and the potential for different outcomes and interpretations of policy decisions. A project to develop a common language to ensure consistency and transparency in modelling research and knowledge translation emerged. Ideally, this common language would reduce variation in results and enhance the application of models in public health. Following the workshop, core members of Pan-InfORM prepared a review of common influenza modelling terms which demonstrated differences, similarities, and discrepancies in terms amongst different studies [18].

After a critical assessment of potential platforms (i.e., virtual or in-person) and current evidence about how and why members engage in CoP, NCCID established mod4PH (Modelling for Public Health) [19], a discussion group on LinkedIn. Mod4PH members are modellers, medical and public health professionals, and researchers from various disciplines. Moderated conversations helped to clarify the use and definitions of disease modelling terms [20]. Building on these discussions, core mod4PH members conducted a comprehensive literature review and following online discussions with the larger group, published a glossary of terms for modelling infectious diseases in 2016 [20].

## 6. Expansion of Pan-InfORM Research Areas

The fifth Pan-InfORM workshop in 2016 had primary objectives of sharing the progress of mod4PH and considering grand challenges of major infectious diseases of on-going concern: tuberculosis (TB), pertussis, and vector-borne diseases [21]. Vaccine hesitancy and modelling immunization to optimize booster schedules were also discussed.

TB was selected as it has been a global public health challenge, defying targets for control set by the World Health Organization [22]. Although Canada is a low incidence country, some northern populations have active TB rates that are more than 300 times that of the Canadian population as a whole, in part due to a history of colonization and resulting inequitable access to care and other resources [23]. Since TB reporting is mandatory, there is a rich source of data for modelling TB in northern populations. This may provide an opportunity to model TB control in partnership with Indigenous communities and provide assessments of potential interventions and their economic feasibility.

The resurgence of Pertussis was selected as another grand challenge because incidence levels in some populations had risen to levels similar to those seen in 1950s. Workshop discussions were an opportunity to evaluate the use of modelling and simulation tools for designing and assessing different vaccine schedules and the cost-effectiveness of program delivery. Modelling priorities for Pertussis included uncertainties about evolving vaccine immunity and identifying optimal booster vaccine schedules.

Strategies for mitigating vector-borne diseases using models were also discussed. Data about vectors (ticks, mosquitoes) were not easy to find or obtain and developing linkable databases was an ongoing challenge. Following the workshop, NCCID and the National Collaborating Centre for Environmental Health (NCCEH) “compiled a list of provincial entomology datasets on vectors”, including the accessibility of data and the type of information each database contains [24]. NCCID also prepared and released a video introducing modelling for public health audiences [25].

## 7. Modelling and health economics

Pan-InfORM's most recent biennial workshop held in 2018 focussed on modelling studies and contributions to the field of emerging infectious diseases, including *Haemophilus influenzae* serotype a (Hia) and *Clostridium difficile*, and determining a roadmap for developing a health economic framework for vaccine evaluation in Canada [26].

Canada has made significant contributions towards vaccine design and production which has been valuable for curtailing the spread of many diseases globally. Vaccine development is dynamic and depends on economic costs, medical needs, timelines, public opinions, and recommendations for policies [26]. Participants acknowledged that Canada does not have a vaccine evaluation framework, but a health economic framework is needed, especially considering increasing demand and growing pipelines for preventative and therapeutic vaccines in the coming future. This framework would provide a platform for further research and application in cost-effectiveness analysis studies and build the capacity to improve program implementation and delivery. Such a framework will require transparency and clarity to be timely, with participation of numerous stakeholders including the National Advisory Committee on Immunization (NACI). Guidance for a framework would include key aspects of health economics, including literature review and the WHO recommendations of cost-effectiveness analysis of vaccines.

Pan-InfORM members also took more concrete steps to develop an Indigenous training program with Indigenous communities to enhance collaborations. Such training could be sowing seeds for enhancing the research capacity for the upcoming generations in Canada and closing knowledge gaps in health research of Indigenous communities. In 2019, Pan-InfORM members and NCCID met with First Nations leaders to outline a summer institute on infectious diseases and public health that would set a foundation for building familiarity with modelling research in communities. The institute was delayed by the COVID-19 pandemic, but introductory videos on modelling for public health for First Nations were completed by NCCID and the NCC for Indigenous Health and shared widely in the summer of 2020 [27],[28].

## 8. Concluding remarks

Mathematical and statistical models are invaluable for designing and evaluating public policy. In times of uncertainty, these models can be used to project the outcomes of different hypotheses, quantify the associated risks, determine the associated economic costs, and provide guidance and recommendations on intervention measures. Despite the breadth of modelling potential prior to the 2003 SARS epidemic in Canada, communication and knowledge translation barriers limited the application of modelling studies in informing major public health decisions. At the time, modelling studies focused on theoretical aspects of complex phenomena with minimal input from end-users. The emergence of SARS was a turning point in the field of disease modelling in Canada as it triggered the development of models with a greater application to public health. However, knowledge translation remained a key challenge in exploiting the full potential of modelling and simulation tools.

Using the CoP model, the Pan-InfORM network bridged many communication gaps and enhanced the utilization of modelling studies in Canadian public health and healthcare system. Pan-InfORM hosted biennial workshops to address public health challenges, develop innovative strategies, and undertake important initiatives to protect communities from persistent and emerging diseases. Notable initiatives catalyzed by Pan-InfORM were: (i) preparedness and evaluation of Canada's response to the 2009 H1N1 pandemic; (ii) established partnerships with Indigenous stakeholders to address unique challenges of vulnerable populations; (iii) the development of a virtual CoP (called mod4PH) to enhance the applicability of disease models, (iv) standardizing a lexicon of disease modelling terms; (v) creating a pathway towards developing a health economic framework for vaccine evaluation in Canada; and (vi) working with Indigenous colleagues to expand knowledge of modelling methods. The achievements and success of this network demonstrate the importance of CoP in disease modelling as a powerful tool in the modern era of global public health.

The Pan-InfORM network continues to work towards its mandate of increasing the use of modelling tools and uptake of research outcomes to inform important public health decisions. During pandemic times, controlling disease spread requires coordinated activities and synergistic efforts at the global scale, and perhaps even the establishment of an international network that uses a similar scientific and knowledge translation approach as Pan-InfORM to guide public policy. This would increase the capacity to optimally respond to emerging health threats by integrating expertise of modellers, policymakers, planners and providers alike to devise innovative, efficient, and feasible intervention measures and protect the global population.
